# Supplementary material for: Isolation and Pathogenic Characterization of Vibrio bivalvicida Associated With a Massive Larval Mortality Event in a Commercial Hatchery of Scallop Argopecten purpuratus in Chile
Source: Front Microbiol. 2019 May 10;10:855. doi: 10.3389/fmicb.2019.00855 (PMC6524457; doi:10.3389/fmicb.2019.00855)

**Supplementary Figure S1.** Phylogenetic tree based on 16S rRNA gene sequence constructed by the neighbour-joining method. Horizontal branch lengths are proportional to evolutionary divergences. Bootstrap values from 1000 replicates appear next to the corresponding branch. *Photobacterium damselae* was used as an outgroup.


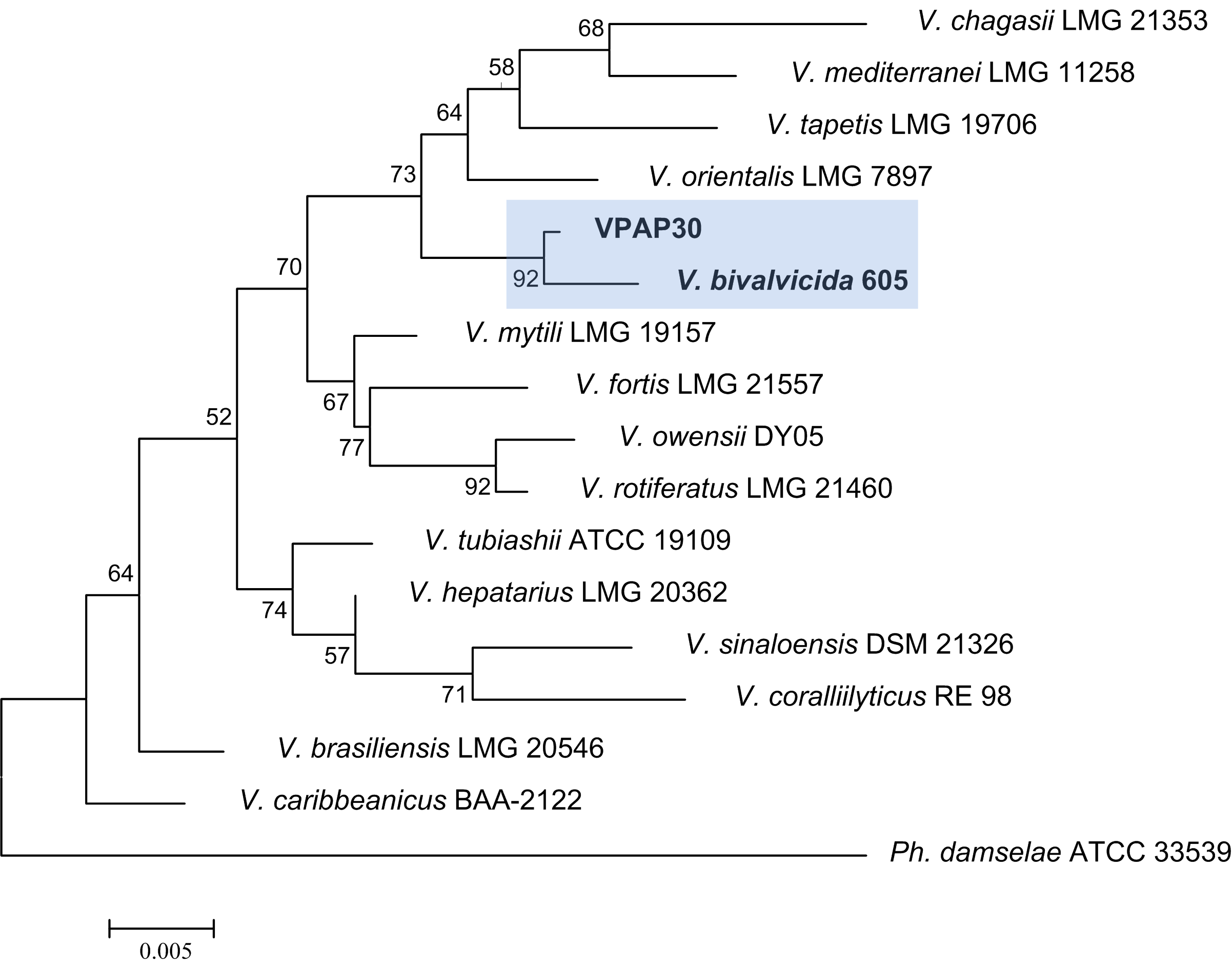

Supplement: Supplementary file 1 [file Table_1.DOCX]
