# Supplementary material for: Isolation and Pathogenic Characterization of Vibrio bivalvicida Associated With a Massive Larval Mortality Event in a Commercial Hatchery of Scallop Argopecten purpuratus in Chile
Source: Front Microbiol. 2019 May 10;10:855. doi: 10.3389/fmicb.2019.00855 (PMC6524457; doi:10.3389/fmicb.2019.00855)
Supplement: Supplementary file 2 [file Table_2.DOCX]

**Supplementary Material 1**

**Fasta sequence of VPAP30 corresponding to the eight housekeeping genes used in the MLSA analysis**

>*fts*Z_VPAP30

TTTTCTTGCAAAGTTGGTGCCGCTTTTTCTTCCACTTTATTGACAGTCTGCTGAGCAGGCGCTGTTTGTGGCTGAGTCTGCGGTTGAGCAACAGGAGCGACTTTCGCTTTGCCACCCGCAACTAAAGTAATATCAGGTTTCTTCTCGTTACCAATACCAGTCGCAACAACTGTCACACGGATTTCATCAGCCATATCAGGATCTAATGAAGTACCGATTACAACGGTTGCGTTATCTGAAGCAAACGCTTTTACTGTGTTACCCACGGTTTCGAATTCATCTAGACGCATATCTAGACCCGCCGTGATGTTAACTAGCACACCGCGTGCTCCAGCAAGATCGATATCTTCTAGAAGTGGGCTAGAAATTGCCATTTCTGCCGCTTCTTCAGCACGATCTTCGCCTTTAGCAACACCGCTACCCATCATGGCATGACCCATTTCTGACATCACTGTTCTTACG

>gapA_VPAP30

AAGACTGTACGTGTTACTGCAGAGCGCAACCCAGAAGACCTAAAGTGGGATGCTATCGATGTTGACGTTGTAGCTGAAGCAACTGGTCTTTTCCTAACTGACGAGACTGCACGTAAGCACATCACTGCTGGTGCAAAGAAAGTTGTTCTTACTGGTCCTTCTAAAGACGCAACTCCAATGTTCGTTATGGGCGTAAACGATTCAACTTACGCTGGTCAAGACATCGTTTCTAACGCTTCTTGTACTACTAACTGTCTAGCGCCTGTAGCTAAAGTTCTTAACGACAAGTTCGGTATCGAATCTGGTCTTATGACTACAGTTCACGCTACTACAGCAACTCAAAAAACTGTAGATGGCCCTTCTGCTAAAGACTGGCGCGGTGGTCGTGGTGCTTCTCAGAACATCATCCCATCTTCAACTGGTGCTGCTAAAGCTGTAGGCGTTGTTCTTCCAGAACTAAACGGCCTTCTAACTGGTATGGCTTTCCGTGTACCAACTGCTAACGTTTCTGTAGTTGACCTAACTGTTAACCTAAAAGAAGCTGCATCTTACGAAGCAATCTGTGCTGCAATGAAAGAAGCTTCTGAAGGCGAGCTAAAAGGTGTTCTAGGTTACACTGAAGACCAAGTTGTTTCTCAAGACTTCATCGGTGAAGTTCAAACTTCAGTATTCGA

>*gyr*B_VPAP30

AAGTGACGCTAACCATCCACCGTGGTGGCCAAATTCATACTCAAACTTACCATCACGGTGAGCCTCAAGCGCCACTATCAGTCGTTGGCGAGACTGAGCAAACAGGTACAGAAATTCGTTTCTGGCCAAGTGAGTTGACTTTCTCTAACACAGAATTCCACTACGACATTCTAGCTAAGCGTCTACGTGAGCTTTCTTTCCTTAACTCTGGCGTATCAATCAAGCTACGTGATGAGCGTGAAGAAGATAAGCAAGATCACTTCATGTATGAAGGTGGTATTCAAGCGTTTGTTGATCACCTAAATACCAACAAAACGCCAATCATCGAGAAAGTATTCCATTTTAACTCTGAGCGTGAAGACGGTATCGCGGTGGAAGTTGCAATGCAGTGGAACGATGGTTACCAAGAGAACATCTTCTGTTTTACTAACAACATCCCTCAGCGTGATGGTGGTACTCACCTTGCCGGTTTCCGTGCAGCCCTTACGCGTACTTTGAACTCGTTCATGGACAAAGAAGGCTTCTCAAAGAAAGCGAAGACCGCGACTTCTGGTGATGATGCACGTGAAGGTCTAACTGCAGTTATTTCAGTTAAGGTTCCTGATCCTAAGTTCTCAAGCCAAACCAAAGACAAACTGGTTTCTTCTGAAGTGAAGTCAGCGGTTGAGCAAGCAATGGGCGAGAAGCTGTCTGAGTTCCTAGTTGAACACCCAGCGGAAGCTAAGACGGTTTGTACTAAGATCATCGATGCAG

>*mre*B_VPAP30

CGTCGCTTCACCAATCAAACTGCCGTAGTTACGACGGACATAGTTGATTATAGCTTCATCAAAACGGTCTCCACCGATACGTACCGACGACGAGTAAACCACACCGTTTAGCGAGATAACTGCCACTTCAGTCGTACCACCGCCGATATCGACCACCATAGAACCTGTTGGCTCTGACACGCGTAAGCCTGCACCAATTGCTGCCGCCATTGGCTCATCAATAAGGTAAACTTCACGAGCCCCCGCACCGAGCGCCGATTCACGAATCGCGCGTCGCTCAACTTGTGTAGAACCACAAGGAACACAAACCAGTACGCGTGGGCTTGGTTTTAGTACGCTGTTGTCATGCACTTGCTTAATAAAGTGCTGAAGCATTTTTTCAGTCACGTAGAAGTCAGCAATAACGCCATCTTTCATTGGACGGATAGCAGAGATGTTCCCCGGTGTACGACCCAACATTTGTTTAGCCGCATGGCCGACAGCCGCAACGCTTTTACCTGCGCGGTTACGGTCTTGGCGAATGGCAACGACTGAAGGCTCGTCTAGAACGATACCTT

>*pyr*H_VPAP30

AATGCTGCCAAATCCATTACTTTTAGTTCTTTTTCAAGAACAGCATTGAATGAAAGCTTATCATACAGCTCTGCGTCTGGGTTTGCTACTGGGTCTGCGGTAAATACACCATCAACTTTTGTCGCTTTTAGAACTACATCCGCTTCAATCTCAATTCCGCGCAAACATGCAGCAGAGTCAGTGGTAAAAAATGGATTCCCCGTACCTGCAGAGAAGATAACCACACGGCCTTGACGTAGTTCGCGAATTGCATCTGCCCAGTTGTAGTCGTCACACACACCCTTAAGAGGAATTGCAGACATTACGCGAGCGTTTACATAAGCACGGTGCAGGGCATCACGCATCGCTAGACCATTCATTACCGTTGCTAGCATCCCCATGTGGTCACCGACAACACGGTTCATACCAGCTTCAGCAAGACCGGCACCACGGAAAAGGTTACCACCACCGATAACCACACCAACTTGAACACCAAGTTCAA

>*rec*A_VPAP30

CCGACAACTTCGTCACCTTCTTTGATAGAACCAGTGCGGCGAATATCAAGACGAACAGATGCGTAGAACTTAAGTGCGTTACCACCCGTGGTTGTTTCAGGGTTACCAAACATCACTCCAATCTTCATACGGATTTGGTTGATGAAGATACACATACAGTTAGATTGCTTTAGGTTACCTGTTAGCTTACGCATAGCTTGAGAAAGCATACGCGCTTGAAGACCCATGTGGCTATCGCCCATTTCACCTTCAATTTCTGCTTTTGGAGTAAGCGCTGCAACCGAGTCGACAACCATTACGTCGATAGCACCTGAACGTGCTAATGCGTCACAAATCTCTAAAGCTTGTTCACCCGTATCTGGCTGAGAAACTAGTAGAGCATCAATATCAACACCTAGCTTCTTAGCATAAACTGGATCAAGTGCGTGTTCCGCATCGATAAAGGCACAAGTTTTACCTTGCTTTTGCGCTGCAGCAATACACTCAAGTGTTAGCGTCGTTTTACCTGATGATTCTGGGCCGTAGATTTCTACGATACGGCCCATCGGAAGACCACCAGCACCTAATGCGATATCCAAAGAAAGTGAACCTGTAGAGATTGTTTCTACATCCATTGCGCGGTTATCACCTAGGCGCATGATTGAACCTTTACCAAATTGCTTTTCAATCTGA

>*rpoA*_VPAP30

GTTAGCACGACTCACGCAAAAGTAACTCTTGAGCCATTAGAGCGTGGTTTTGGCCATACTCTTGGTAATGCACTTCGCCGTATTCTTCTATCTTCTATGCCAGGTTGTGCTGTAACAGAAGTAGAAATCGAAGGCGTACTTCATGAGTACAGCACTAAAGAAGGCGTTCAAGAAGATATTCTTGAAATCCTACTTAACCTTAAAGGTTTAGCTGTACGCGTTGCCGAAGGCAAAGATGAAGTGTTCATTACTTTGAACAAATCAGGCTCGGGCCCTGTGGTTGCAGGTGACATCACCCATGATGGTGATGTAGAGATCGCTAACCCTGAACACGTTGTTTGTCACCTAACAGATGACAACGCTGAGATCGCAATGCGTATCAAAGTTGAACGTGGTCGTGGTTATGTTCCAGCTTCGGCTCGTATCCATACTGAAGAAGATGAGCGTCCAATTGGTCGCCTACTGGTTGACGCTACGTACAGTCCAGTAGACAAGATTGCTTACTCTGTTGAAGCAGCTCGTGTAGAGCAGCGTACAGACTTAGACAAGCTTGTTATCGATATGGAAACGAACGGTACTCTAGACCCTGAGGAAGCAATCCGTCGTGCAGCTACTATCCTAGCTGAGCAATTGGATGCGTTCGTAGATCTTCGTGATGTACGTGTACCTGAGGAGAAGGAAGAGAAGCCAGAATTCGATCCGATCCTACTGCGTCCTGTAGACGATCTTGAACTAACAGTTCGCTCTGCTAACTGTCTGAAAGCAGAAGCGATTCACTACATCGGTGATCTTGTACAGCGTACTGAGGTTGAGCTACTTAAAACGCCTAACCTTGGTAAAAAATCTCTT

>*topA*_VPAP30

CCAAAAACGCTATCCAACAGGCGTTCGAAAAACCGGGCGAGCTGAGTATGGATGGCGTTAATGCCCAGCAAGCACGTCGTTTTATGGACCGTGTGGTTGGCTTTATGGTTTCTCCACTGTTATGGAAGAAAGTGGCACGTGGTTTGTCTGCAGGTCGTGTTCAATCGGTCGCTGTAAAATTACTTGTTGAGCGTGAACGTGAAATTAAAGCGTTTATCCCTGAAGAGTTCTGGGATATTCATGCTGATACCAAAACTCAAGACAAAACTGATTTCCGTTTGCAAGTGGCGCAGAAAGAAGGCGTCGCTTTTAAGCCTGTTAACGAAGCAGAAACGAAATCTGCAATGGCTGTGCTTGAAAATGCACGCTATGAAGTGTGTAAGCGTGAAGACCGTCCAACATCCAGCAAGCCGTCTGCACCGTTTATCACGTCAACTCTGCAGCAAGCAGCGAGTACTCGTTTAGGTTACGGCGTTAAAAAAACCATGATGTTAGCTCAGCGTCTCTATGAAGCGGGCTACATCACCTATATGCGTACTGACTCGACAAACCTAAGTGCTGAAGCTGTAGAGACCGTACGTGGCTTTATCGGTAGCGAGTATGGTGAGGCGTACCTTCCAGGTAA
