# Supplementary material for: Isolation and Pathogenic Characterization of Vibrio bivalvicida Associated With a Massive Larval Mortality Event in a Commercial Hatchery of Scallop Argopecten purpuratus in Chile
Source: Front Microbiol. 2019 May 10;10:855. doi: 10.3389/fmicb.2019.00855 (PMC6524457; doi:10.3389/fmicb.2019.00855)
Supplement: Supplementary file 3 [file Table_3.docx]

**Table 1.** List of strains and sequences accession numbers used for the MLSA.

| **Species** | Strain | *fts*Z | *gap*A | *gyr*B | *mre*B | *pyr*H | *rec*A | *top*A | *rpo*A |
| --- | --- | --- | --- | --- | --- | --- | --- | --- | --- |
| ***V. crosai*** | CAIM 1437^T^ | KC774631 | KC774632 | LN831190 | LT594322 | KC774630 | KC774629 | KC774628 | LN831191 |
| ***V. ichthyoenteri*** | LMG19664^T^=ATCC 700023^T^ | DQ907354 | DQ907287 | HM771365 | DQ907426 | HM771375 | AJ842446 | DQ907493 | AJ842633 |
| ***V. scophthalmi*** | LMG 19158^T^ | HM771356 | HM771361 | HM771366 | HM771371 | HM771376 | HM771381 | HM771335 | HM771386 |
| ***V. nereis*** | LMG 3895^T^ | DQ907362 | DQ449617 | AB298235 | DQ907436 | JN968379 | AJ842479 | DQ907504 | AJ842666 |
| ***V. xuii*** | LMG 21346^T^ | DQ907384 | DQ907315 | AB298254 | DQ907456 | GU266284 | AJ842529 | DQ907524 | AJ842742 |
| ***V. caribbeanicus*** | ATCC BAA-2122^T^ | HM771358 | HM771363 | HM771368 | HM771373 | HM771378 | HM771383 | HM771337 | HM771388 |
| ***V. pectenicida*** | LMG 19642^T^= CIP 105190^T^ | DQ907368 | DQ907301 | AB298240 | DQ907441 | JN039143 | JN039141 | DQ907510 | JN039142 |
| ***V. coralliilyticus*** | LMG 20984^T^ | DQ907341 | DQ907279 | AB298210 | DQ907412 | GU266292 | AJ842402 | EF114213 | AJ842587 |
| ***V. neptunius*** | LMG 20536^T^ | DQ907361 | DQ907296 | AB298234 | DQ907435 | JN039151 | JN039152 | DQ907503 | JN039153 |
| ***V. brasiliensis*** | LMG 20546^T^ | HM771354 | HM771359 | HM771364 | HM771369 | HM771374 | HM771379 | HM771333 | HM771384 |
| ***V. tubiashii*** | ATCC 19109^T^ | CP009354, CP009355, CP009356, CP009357, CP009358, CP009359 (WGS data) | | | | | | | |
| ***V. sinaloensis*** | LMG 25238^T^= DSM 21326^T^ | HM771357 | HM771362 | HM771367 | HM771372 | HM771377 | JN039138 | HM771336 | JN039159 |
| ***V. orientalis*** | LMG 7897^T^= ATCC 33934^T^ | DQ907365 | DQ907299 | EF380260 | DQ907439 | EU118243 | AJ842485 | DQ907507 | AJ842672 |
| ***V. hepatarius*** | LMG 20362^T^ | DQ907352 | DQ907285 | AB298222 | DQ907424 | JF316674 | AJ842444 | DQ907491 | AJ842631 |
| ***V. europaeus*** | PP-638^T^ | LUAX00000000 (WGS data) | | | | | | | |
| ***V. bivalvicida*** | 605^T^ | LLEI00000000 (WGS data) | | | | | | | |
| ***V. bivalvicida*** | VPAP30 | LBLS00000000 (WGS data) | | | | | | | |
| ***Ph. damselae* subsp*. damselae*** | ATCC 33539^T^=LMG 7892^T^ | DQ907319 | DQ907258 | AB298188 | DQ907386 | EF380236 | AJ842357 | DQ907458 | AJ842541 |

**Supplemental Table 2**. Metabolic profile of *V. bivalvicida* VPAP30 strain determinate using the Biolog system (Biolog Inc.).

| Carbon source | Result |  | Carbon source | Result |  | Carbon source | Result |
| --- | --- | --- | --- | --- | --- | --- | --- |
|  |  |  |  |  |  |  |  |
| α-cyclodextrin | − |  | Xylitol | − |  | L-alanine | + |
| Dextrin | + |  | Methyl-pyruvate | − |  | L-alanyl-glycine | + |
| Glycogen | + |  | Mono-methyl-succinate | − |  | L-asparagine | + |
| Tween 40 | +^w^ |  | Acetic acid | +^w^ |  | L-aspartic acid | + |
| Tween 80 | +^w^ |  | Cis-aconitic acid | − |  | L-glutamic acid | + |
| N-acetyl-D-galactosamine | − |  | Citric acid | − |  | Glycyl L-aspartic acid | + |
| N-acetyl-D-glucosamine | + |  | Formic acid | − |  | Glycyl L-glutamic acid | + |
| Adonitol | − |  | D-galactonic acid lactona | − |  | L-histidine | +^w^ |
| L-arabinose | − |  | D-galacturonic acid | − |  | Hydroxy L-proline | − |
| D-arabinol | − |  | D-gluconic acid | − |  | L-leucine | − |
| D-cellobiose | + |  | D-glucosaminic acid | − |  | L-ornithine | +^w^ |
| i-erythritol | − |  | D-glucuronic acid | − |  | L-phenilalanine | − |
| D-fructose | + |  | α-hydroxy butyric acid | − |  | L-proline | + |
| L-fucose | − |  | β-hydroxy butyric acid | +^w^ |  | L-pyroglutamic acid | − |
| D-galactose | − |  | γ-hydroxy butyric acid | − |  | D-serine | − |
| Gentiobiose | − |  | p-hydroxy phenylacetic acid | − |  | L-serine | + |
| α-D-glucose | + |  | Itaconic acid | − |  | L-threonine | + |
| m-inositol | − |  | α-keto butyric acid | +^w^ |  | D,L-carnitine | − |
| α-D-lactose | − |  | α-keto glutaric acid | − |  | γ-amino butyric acid | − |
| Lactulose | − |  | α-keto valeric acid | − |  | Urocanic acid | − |
| Maltose | + |  | D,L-lactic acid | + |  | Inosine | + |
| D-mannitol | - |  | Malonic acid | − |  | Uridine | + |
| D-mannose | + |  | Propionic acid | − |  | Thymidine | + |
| D-melibiose | + |  | Quinic acid | − |  | Phenyethylamine | − |
| β-metil-D-glucoside | − |  | D-saccharic acid | − |  | Putrescine | − |
| D-psicose | − |  | Sebacic acid | − |  | 2-aminoethanol | − |
| D-raffinose | − |  | Succinic acid | + |  | 2,3-butanediol | − |
| L-rhamnosa | − |  | Bromo succinic acid | + |  | Glycerol | + |
| D-sorbitol | − |  | Succinamic acid | − |  | D,L α-glycerol phosphate | − |
| Sucrose | + |  | Glucuronamide | − |  | Glucose 1-phosphate | − |
| D-trehalose | + |  | L-alaninamide | − |  | Glucose 6-phosphate | − |
| Turanose | − |  | D-alanine | − |  |  |  |
|  |  |  |  |  |  |  |  |

+: Positive reaction; +^w^: Weak reaction; −: Negative reaction
